# Association of the Estrogen Receptor 1 (ESR1) Gene with Body Height in Adult Males from Two Swedish Population Cohorts

**DOI:** 10.1371/journal.pone.0001807

**Published:** 2008-03-19

**Authors:** Andreas Dahlgren, Per Lundmark, Tomas Axelsson, Lars Lind, Ann-Christine Syvänen

**Affiliations:** 1 Molecular Medicine, Department of Medical Sciences, Uppsala University, Uppsala, Sweden; 2 Department of Medical Sciences, Uppsala University Hospital, Uppsala, Sweden; Freie Universitaet Berlin, Germany

## Abstract

Human body height is a complex genetic trait with high heritability. We performed an association study of 17 candidate genes for height in the Uppsala Longitudinal Study of Adult Men (ULSAM) that consists of 1153 elderly men of age 70 born in the central region of Sweden. First we genotyped a panel of 137 single nucleotide polymorphism (SNPs) evenly distributed across the candidate genes in the ULSAM cohort. We identified 4 SNPs in the estrogen receptor gene (ESR1) on chromosome 6q25.1 with suggestive signals of association (p<0.05) with standing body height. This result was followed up by genotyping the same 25 SNPs in the ESR1 gene as in ULSAM in a second population cohort, the Prospective Investigation of the Vasculature in Uppsala Seniors (PIVUS) cohort that consist of 507 males and 509 females of age 70 from the same geographical region as ULSAM. One SNP, rs2179922 located in intron 4 of ESR1 showed and association signal (p = 0.0056) in the male samples from the PIVUS cohort. Homozygote carriers of the G-allele of the SNP rs2179922 were on average 0.90 cm taller than individuals with the two other genotypes at this SNP in the ULSAM cohort and 2.3 cm taller in the PIVUS cohort. No association was observed for the females in the PIVUS cohort.

## Introduction

Most human traits and diseases are complex, involving multiple genes and environmental factors. Human body height and its variation between individuals is a typical complex trait with high heritability [1]. Several genome-wide linkage studies on height have been performed in recent years, and these studies suggest multiple genomic regions harboring genes affecting body height [2]–[9]. Loci with suggested linkage with body height have been reported on all chromosomes except chromosomes 10, 16 and 19, but only a subset of these loci have been identified in multiple studies. This lack of reproducible findings could be due to a large number of genes with small individual additive effects on height. This notion is corroborated by a recent study that studied genome partitioning of genetic variation using linkage data from over ten thousand sib pairs and found that most chromosomes could harbor loci that affect human height[10]. Given that most population-based studies have collected information on anthropometric measures, including height, there is a surprising lack of findings of genes that are associated with normal body height in populations. However, a recent genome-wide association study identified a common variant of the high mobility group-A2 gene (HMGA2) that shows convincing evidence of association with height, and replicated this finding in several population cohorts[11]. In the current study we analyzed the population-based Uppsala Longitudinal Study of Adult Men (ULSAM) cohort for the association of single nucleotide polymorphisms (SNPs) in 17 candidate genes with height (Table 1), and followed up our findings of suggestive association signals in the estrogen receptor gene (ESR1) in a second very similar population cohort, the Prospective Investigation of the Vasculature in Uppsala Seniors (PIVUS) cohort.

**Table 1:** Genes and number of SNPs analyzed for association with body height

Gene symbol	Gene name	Chromosome position	Number of SNPs analyzed
BRD8	bromodomain containing 8	5q31.2	7
COMP	cartilage oligomeric matrix protein	19p13.11	3
DRD2	dopamine receptor D2	11q23.2	8
EGR3	early growth response 3	8p21.3	5
ESR1	estrogen receptor 1	6q25.1	23
FGF13	fibroblast growth factor 13	Xq26.3	7
FGFR3	fibroblast growth factor receptor 3	4p16.3	3
GH1	growth hormone 1	17q23.3	3
GHR	growth hormone receptor	5p12	20
GHRH	growth hormone releasing hormone	20q11.23	5
GHRHR	growth hormone releasing hormone receptor	7p14.3	6
GRB2	growth factor receptor-bound protein 2	17q25.1	7
IRS4	insulin receptor substrate 4	Xq22.3	2
MMP16	matrix metallopeptidase 16 (membrane-inserted)	8q21.3	21
MMP19	matrix metallopeptidase 19	12q13.2	6
PHACTR2	phosphatase and actin regulator 2	6q24.2	8
SOX3	SRY (sex determining region Y)-box 3	Xq27.1	3

## Materials and Methods

### Study cohorts

ULSAM is an on-going, longitudinal, epidemiologic study on cardiovascular diseases in all men born between 1920 and 1924 in Uppsala County, Sweden (www.pubcare.uu.se/ULSAM) [12]. The men were investigated at ages 50, 60, 70, 77 and 82. The DNA samples analyzed in the present study (n = 1153) were collected when the participants had reached 70 years of age and standing body height used in the current study was measured at the same time. PIVUS is also a longitudinal population-based study focused on vascular function and body composition (www.medsci.uu.se/pivus/pivus.htm). The PIVUS cohort consists of 1016 participants with 507 males and 509 females of age 70 born in 1931 or later [13]. Measurements of standing body height were taken at the first sample collection at age 70. Height was measured to the nearest cm in both cohorts. Both population cohorts are from the Uppsala region in Central Sweden. The studies were approved by the Ethics Committee of the Faculty of Medicine at Uppsala University and informed consent was obtained from all participants.

### Genotyping

A panel of 174 single nucleotide polymorphisms (SNP) located in 17 potential candidate genes for height were selected from dbSNP (www.ncbi.nlm.nih.gov/projects/SNP/) for genotyping in the DNA samples from the ULSAM cohorts. The principle for SNP selection was to cover the genes, including exons and introns using SNPs with minor allele frequencies >0.05 at an even spacing of 1.5 kb to 12.5 kb, depending in the size of the genes. In addition, an Illumina design score of 0.5 was used as the lower limit for acceptance of a SNP for genotyping. The 174 selected SNPs (Supplementary Table S1) were genotyped in the ULSAM cohort using the GoldenGate assay [14] and the Illumina BeadArray system (Illumina, San Diego, CA, USA). Genotypes were successfully called for 137 of the selected SNPs. Out of the 37 SNPs that were failed, twenty five were not polymorphic, ten SNPs had sample call rates below 90% and finally two SNPs for which the genotype distribution deviated from Hardy-Weinberg equilibrium (p>0.05) were excluded from further analysis. The average call rate for the SNPs that passed quality control was 96.3% and the reproducibility of genotyping was 99.98% according duplicate determination of 4% of the genotypes. The rs-numbers and nucleotide positions for the successfully genotyped SNPs are provided in the Appendix. An additional panel of 33 tag-SNPs in the *ESR1* gene were selected using the Haploview software [15] by considering the variation captured by the 25 successfully genotyped SNPs in the *ESR1* gene. In addition to the 33 tag-SNPs, three SNPs successfully genotyped with the GoldenGate assay were included for testing accuracy between genotyping methods. The second round of *ESR1* genotyping the ULSAM cohort was performed using the SNPstream™ genotyping system (Beckman Coulter, Fullerton, CA, USA) [16]. Of the 36 SNPs, 24 passed quality control. The average call rate was 92% and the accuracy of the genotyping was 99.7% based on comparison of the data from the 3 SNPs genotyped by both systems. In total 47 SNPs in the ESR1 gene were successfully genotyped in the ULSAM cohort (Table 2). Twenty five SNPs in the *ERS1* gene that were genotyped in the ULSAM cohort were also genotyped in the PIVUS cohort using the GoldenGate Assay. All SNPs were successfully genotyped with an average call rate of 99.5% and reproducibility of 99.8% based on duplication of 2% of the genotypes.

**Table 2:** SNPs genotyped in the estrogen receptor alpha gene

		ULSAM		PIVUS		
SNP	SNP-allele major/minor	MAF^1^	p-value	MAF	p-value	Genotyping method^2^
rs10484922	C/T	0.11	0.93	-	-	3
rs3853248	T/C	0.13	0.62	0.15	0.57	1,2
rs11155813	T/C	0.12	0.47	0.13	0.86	1,2
rs827423	A/G	0.48	0.014	-	-	3
rs9322331	C/T	0.31	0.040	-	-	3
rs2234693	T/C	0.43	0.044	0.46	0.95	1,2,3
rs1709183	T/C	0.28	0.54	0.27	0.35	1,2
rs11155819	T/C	0.33	0.15	-	-	3
rs9340835	G/A	0.33	0.092	0.34	0.28	1,2
rs9322335	C/T	0.25	0.026	-	-	3
rs1913474	G/A	0.23	0.37	0.22	0.71	1,2,3
rs2347867	A/G	0.33	0.037	0.32	0.44	1,2
rs6557171	C/T	0.30	0.12	0.29	0.22	1,2
rs12204714	T/C	0.33	0.068	-	-	3
rs988328	T/C	0.14	0.24	-	-	3
rs6927072	G/T	0.33	0.023	0.31	0.18	1,2
rs1801132	C/G	0.23	0.077	0.20	0.46	1,2
rs3020314	T/C	0.33	0.29	-	-	3
rs3020377	A/G	0.36	0.11	0.31	0.11	1,2
rs3020393	A/G	0.17	0.17	-	-	3
rs3020394	A/G	0.32	0.21	-	-	3
rs3003925	A/G	0.21	0.35	-	-	3
rs6557177	T/C	0.15	0.50	-	-	3
rs985694	C/T	0.19	0.10	0.17	0.016	1,2
rs1884049	C/T	0.19	0.023	-	-	3
rs2179922	G/A	0.11	0.020	0.10	0.0056	1,2
rs2982683	C/T	0.30	0.26	-	-	3
rs3020407	A/G	0.34	0.10	-	-	3
rs7754762	T/A	0.13	0.24	0.13	0.30	1
rs722208	A/G	0.31	0.47	0.31	0.33	1,2
rs2207232	T/C	0.13	0.22	0.13	0.33	1,2,3
rs3020422	G/A	0.37	0.48	0.40	0.44	1,2
rs3020353	C/A	-	-	0.40	0.44	2
rs9371573	C/A	0.37	0.35	-	-	1
rs3020368	C/T	0.090	1.0	0.092	0.71	1,2
rs3778082	G/A	0.14	0.18	0.14	0.22	1,2
rs11965894	C/T	0.090	0.38	-	-	3
rs7766585	T/G	0.15	0.076	0.12	0.20	1,2
rs9322355	C/T	0.10	0.18	0.11	0.28	1,2
rs3020382	T/C	0.18	0.58	-	-	3
rs2813544	A/G	0.20	0.87	0.20	0.80	1,2
rs7450824	T/C	0.26	0.39	-	-	1
rs2747653	T/C	0.20	0.47	-	-	3
rs2813563	C/T	0.21	0.48	-	-	3
rs725467	C/T	0.20	0.78	-	-	1,2
rs2673790	A/G	0.29	0.33	-	-	3
rs2252837	C/T	0.29	0.64	-	-	3

SNPs are listed 5′-3′ from top to bottom of this table.

1 Minor allele frequency.

2 1: GoldenGate™ assay ULSAM panel, 2: GoldenGate™ assay PIVUS panel, 3: SNPstream™ system.

### Statistical analysis

**Figure 1:**
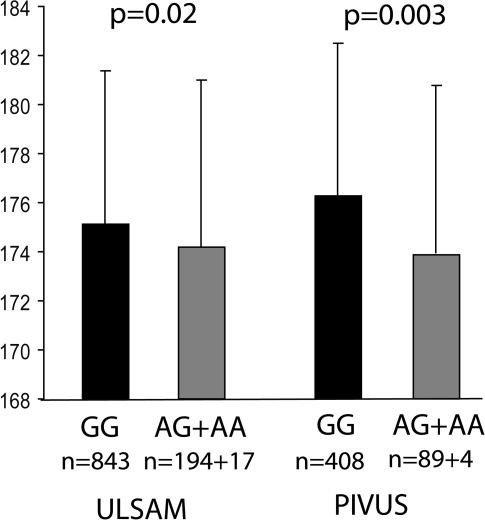
Analysis of body height according to genotype of the SNP rs2179922 in the ULSAM and PIVUS cohorts. The number of observations (n) is shown below each genotype group and the error bars indicate the standard deviation in cm in each group. Mean body height in the ULSAM cohort was 175.1±6.0 cm for GG genotype group and 174.2±6.8 cm for the AG+AA group. Mean body height in the PIVUS cohort was 172.2±6.3 cm for GG genotype group and 173.9±6.8 cm for the AG+AA group.

The statistical analysis was performed using the free statistical software environment “R” [17]. Analysis of variance (ANOVA) was performed to test for association between SNPs and height. Replicated nominally significant results from ANOVA (p<0.05) were tested using a two sample t-test on body height according to genotype distribution (Figure 1). In the PIVUS cohort, males and females were analyzed separately because of known differences in heritability of body height [1]. The Haploview software “Tagger” was used for SNP selection and to estimate the amount of SNP variation captured by the panel of SNPs.

## Results

**Figure 2:**
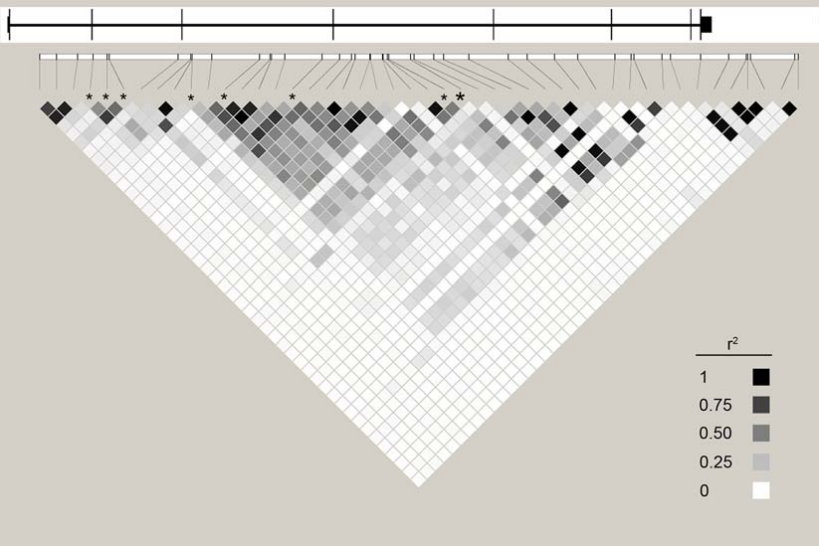
Schematic illustration of the estrogen receptor gene (*ESR1*) and its linkage disequilibrium structure. The line in the top part of the figure illustrates the ESR1 gene with the exons marked with horizontal lines. The lower part of the figure shows the positions of the single nucleotide polymorphisms (SNPs) in *ESR1* genotyped in the ULSAM cohort with pair-wise linkage disequilibrium (r^2^) values. The SNPs with nominal p-values<0.05 in the ULSAM cohort are marked with stars, and the SNP rs2179922 located in intron 4 of ESR1 with p = 0.0056 in the PIVUS cohort is marked by a large star.

The initial screening phase of our study included 17 candidate genes for height. Three main criteria were used for selecting the genes for study. The BRD8, ESR1, GHRH, MMP16, MMP19, and PHACTR2 genes were primarily selected based on their position in a genomic region identified by a genome-wide linkage study for height, in combination with a possible functional role in growth and bone development. The COMP, DRD2, EGR3, GH1, GHR, GHRHR and GRB2 genes were selected solely according to their known biological functions related to growth and bone development. The FGF13, FGFR3, IRS4 and SOX3 are genes reported to be involved in rare syndromes characterized by short stature (Table 1). A panel of SNPs that were evenly distributed across these genes was genotyped in DNA samples from 1153 males of age 70 from the population-based ULSAM cohort of elderly men. Using ANOVA to analyze the SNPs for association with standing body height, we observed 4 SNPs (rs2234693, rs2347867, rs6927072, rs2179922) with nominally significant association signals (p<0.05) in the estrogen receptor gene (*ESR1*) (Table 2). We did not apply correction for multiple testing in this exploratory screen because of the availability of the highly similar PIVUS cohort for replication. Based on the suggestive findings in the ULSAM cohort, we selected the *ESR1* gene for further study. By analyzing the data from the same set of 25 SNPs in the *ESR1* gene for association with height in the PIVUS cohort, we observed a clear association signal from the SNP rs2179922 located in intron 4 of *ESR1* in males from the PIVUS cohort (p = 0.0056) (Table 2). No association was observed for the females from the PIVUS cohort. Homozygote carriers of the G-allele were on average 0.90 cm taller than individuals with the two other genotypes at this SNP in the ULSAM cohort (Figure 1), and 2.3 cm taller in the PIVUS cohort. To complement the variation of the *ESR1* gene captured by the original set of 25 SNPs, we genotyped an additional panel of 21 tag-SNPs in the ULSAM cohort. Three of them yielded associations signals with nominal p-values<0.05 (Table 2), but addition of these 21 SNPs did not strengthen our previous findings in the ULSAM cohort notably. Figure 2 shows the positions of the 47 SNPs in the *ESR1* gene that were genotyped in the ULSAM cohort and the linkage disequilibrium values between the SNPs. These SNPs capture 73% of the common SNP variation of *ESR1* (MAF≥0.05) in the European sample from the HapMap project.

## Discussion

In our study we screened potential candidate genes for body height and identified and replicated association of the SNP rs2179922 in intron 4 of the *ESR1* gene with standing body height in elderly males from two distinct, but highly similar Swedish population cohorts. The *ESR1* gene is located at chromosome 6q25.1, which is a region that has been linked with body height in two large genome-wide linkage studies [2], [18]. Estrogen plays an important role in the regulation of bone maturation and in the closure of epiphyseal plates in growing bones in both females and males [19]. The role for estrogen in skeletal development and growth in females has been well established since the 50s. More recently it was recognized that estrogen also affects bone development and body height in males [20]. This notion was pioneered by a case report of a male with complete estrogen resistance due to homozygous mutations in the *ESR1* gene [21]. This patient showed incomplete epiphyseal closure and a history of continued growth into adulthood resulting in a standing body height of 204 cm. Based on this strong effect of rare recessive mutations in the *ESR1* on body height, it is conceivable that other, more common variants of *ESR1* with smaller effects could affect body height on the population level. Given the importance of estrogen for adult height in both genders, and two previous reports that suggested associations between SNPs in the *ESR1* gene and adult body height in females [22], [23], it was surprising that we did not detect an association between the *ESR1* and height in the females from the PIVUS cohort. In previous studies a haplotype of *ESR1* defined by the SNP rs2234693 was associated with height in females and young boys [23], [24]. In our study we also genotyped the SNP rs2234693, and it showed a suggestive association with height in the ULSAM cohort (p = 0.04), but this finding did not replicate in males or females from the PIVUS cohort. In our study samples, the SNPs rs2234693 is not in LD (r^2^ = 0) with the SNP rs2179922, which showed association with height in males from both the ULSAM and PIVUS cohorts. The most plausible explanation for the lack of association between *ESR1* and height in females in our study is that we did not have sufficient statistical power to detect weak genetic effects. The effects on body height between different variants of the *ESR1* gene may also differ between males and females. The effect on body height of the G-allele of the SNP rs2179922 in *ESR1* that we observed in males from the ULSAM and PIVUS cohorts is comparable to the effect of the SNP rs1042725 in the HMGA2 gene in adult males in multiple cohorts from the UK and Sweden [11].

In conclusion, the results from our study on informative common SNPs in 17 strong candidate genes for height are in line with previous studies, which indicate that a large number of genetic variants with small individual effects contribute to the high heritability of human body height. Our results suggest that *ESR1* is one of these genes, but the power of our study does not allow us to exclude small effects on height by the other candidate genes analyzed. To identify the actual functional variants in the *ESR1* gene that affect height will require re-sequencing of the genes to identify possible rare variants, and functional studies on the molecular level, as well as very large population-based studies on the interactions between genes and with factors from the environment.

## Supporting Information

Table S1 Gene, chromosome and nucleotide position for the SNPs analyzed in the ULSAM Illumina panel.
(0.03 MB XLS)
